# Seneca Valley Virus 3C^pro^ Mediates Cleavage and Redistribution of Nucleolin To Facilitate Viral Replication

**DOI:** 10.1128/spectrum.00304-22

**Published:** 2022-03-31

**Authors:** Jiangwei Song, Rong Quan, Dan Wang, Jue Liu

**Affiliations:** a Beijing Key Laboratory for Prevention and Control of Infectious Diseases in Livestock and Poultry, Institute of Animal Husbandry and Veterinary Medicine, Beijing Academy of Agriculture and Forestry Sciences, Beijing, China; b College of Veterinary Medicine, Yangzhou University, Yangzhou, Jiangsu Province, China; c Jiangsu Co-innovation Center for Prevention and Control of Important Animal Infectious Diseases and Zoonoses, Yangzhou University, Yangzhou, Jiangsu Province, China; Changchun Veterinary Research Institute

**Keywords:** Seneca Valley virus (SVV), nucleolin (NCL), cleavage, 3C protease (3C^pro^), replication

## Abstract

Seneca Valley virus (SVV) is a recently discovered pathogen that poses a significant threat to the global pig industry. It has been shown that many viruses are reliant on nucleocytoplasmic trafficking of nucleolin (NCL) for their own replication. Here, we demonstrate that NCL, a critical protein component of the nucleolus, is cleaved and translocated out of the nucleoli following SVV infection. Furthermore, our data suggest that SVV 3C protease (3C^pro^) is responsible for this cleavage and subsequent delocalization from the nucleoli, and that inactivation of this protease activity abolished this cleavage and translocation. SVV 3C^pro^ cleaved NCL at residue Q545, and the cleavage fragment (aa 1 to 545) facilitated viral replication, which was similar to the activities described for full-length NCL. Small interfering RNA-mediated knockdown indicated that NCL is required for efficient viral replication and viral protein expression. In contrast, lentivirus-mediated overexpression of NCL significantly enhanced viral replication. Taken together, these results indicate that SVV 3C^pro^ targets NCL for its cleavage and redistribution, which contributes to efficient viral replication, thereby emphasizing the potential target of antiviral strategies for the control of SVV infection.

**IMPORTANCE** The nucleolus is a subnuclear cellular compartment, and nucleolin (NCL) resides predominantly in the nucleolus. NCL participates in viral replication, translation, internalization, and also serves as a receptor for virus entry. The interaction between NCL and SVV is still unknown. Here, we demonstrate that SVV 3C^pro^ targets NCL for its cleavage and nucleocytoplasmic transportation, which contributes to efficient viral replication. Our results reveal novel function of SVV 3C^pro^ and provide further insight into the mechanisms by which SVV utilizes nucleoli for efficient replication.

## INTRODUCTION

Seneca Valley virus (SVV) was first identified as a cell culture contaminant in the United States in 2002 (1, 2) and has since been identified as the etiological agent of serious outbreaks of vesicular disease in pigs from around the world (3–9). Typical clinicopathological signs include vesicular lesions in the oral passages, snout, hooves, and coronary band, leading to significant economic consequences (2). Importantly, SVV-induced vesicular disease is indistinguishable from foot-and-mouth disease (FMD) and other swine vesicular diseases. Guangdong province was the first Chinese province to report a potential SVV outbreak in 2015 (3) with an increasing number of outbreaks being reported ever since (10).

SVV is a non-enveloped single-stranded RNA virus within the *Senecavirus* genus of the family *Picornaviridae* (1, 11). The SVV genome comprises approximately 7 200 bases containing one open reading frame that is translated into a polyprotein, which is cleaved into four viral capsid proteins and eight non-structural proteins (1). Non-structural protein 3C proteinase (3C^pro^) is a cysteine protease encoding a catalytic box histidine (40) and (160) were catalytic residues in 3C^pro^ which are both indispensable for the direct cleavage and degradation of many host proteins (1, 12–15). SVV 3C^pro^ targets critical cellular proteins for cleavage to inhibit host innate immune responses, including the mitochondrial antiviral signaling protein, Toll/interleukin 1 (IL-1) receptor domain-containing adaptor inducing protein, and TRAF family member-associated NF-κB activator (12). Moreover, 3C^pro^ degrades retinoic acid-inducible gene I (RIG-I) to inhibit type I interferon production (14) and SVV 3C^pro^ cleaves selective autophagy receptor SQSTM1/p62 (sequestosome 1), which inhibits SVV replication and reduces selective autophagy (16, 17). 3C^pro^ also cleaves NLRP3, which may inhibit caspase-1 activation to facilitate 3C^pro^-specific cleavage of porcine gasdermin D (GSDMD) at its target site close to the caspase-1 GSDMD cleavage site (18). This cleaved GSDMD has a similar capacity to induce cell death as the native caspase-1 GSDMD cleavage product (18). In addition, 3C^pro^ activity can also induce apoptosis by activating caspase-3, -8, and -9, or by cleaving NF-κB-p65 and poly (ADPribose) polymerase (19). 3C^pro^ also cleaves poly(A)-binding protein cytoplasmic 1 to facilitate SVV replication (13). SVV infection induces stress granule (SG) formation via the PKR-eIF2α signaling pathway, and 3C^pro^ has been shown to inhibit SG formation by disrupting eIF4GI/G3BP1 interaction (20).

Nucleolin (NCL) is distributed across the cell surface and in the cytoplasm, and resides predominantly in the nucleolus, where it performs several unique functions. NCL participates in diverse cellular processes, including RNA transcription, ribosome biogenesis, nucleocytoplasmic transport, and posttranscriptional regulation of mRNAs (21–27). NCL is also associated with proliferation of many viruses with the nucleocytoplasmic redistribution of this protein known to play an important role in viral replication (28–30). For example, NCL interacts with the 3′-UTR of poliovirus to promote translation of viral RNA (30) while a more recent study reported that NCL interacts with the internal ribosome entry site (IRES) of food and mouth disease (FMDV), which promotes IRES-driven translation of FMDV RNA via its active recruitment of translation initiation complexes (29). NCL also interacts with the US11 protein from the herpes simplex virus 1 (HSV-1) and is closely associated with its nucleocytoplasmic transport (28). NCL has also been shown to interact with the rabbit hemorrhagic disease virus (RHDV) capsid protein and mediates the internalization of RHDV through clathrin-dependent endocytosis (31). In addition, cell surface NCL serves as a receptor for human respiratory syncytial virus (RSV) (32), adeno-associated virus type-2 (33), coxsackie B virus (34), and human immunodeficiency virus type 1 (HIV-1) (35), and NCL directly interacts with the VP1 capsid protein of enterovirus 71 (EV71) promoting EV71 binding, infection, and production (36).

Our data show that SVV infection upregulated the expression of NCL and induced NCL cleavage. In addition, SVV infection induced cytoplasmic redistribution of NCL from the nucleus and we found that the cleavage and redistribution of NCL was modulated by the protease activity of 3C^pro^. SVV 3C^pro^ cleaved NCL at residue Q545, and this cleaved NCL then facilitated viral replication. Knockdown of NCL expression also dramatically inhibited SVV replication, while its overexpression promoted SVV replication. Collectively, these findings indicate that NCL contributes to SVV propagation through its cleavage and translocation which is mediated by its 3C^pro^ protein.

## RESULTS

### SVV infection cleaves and upregulates NCL expression.

**FIG 1:**
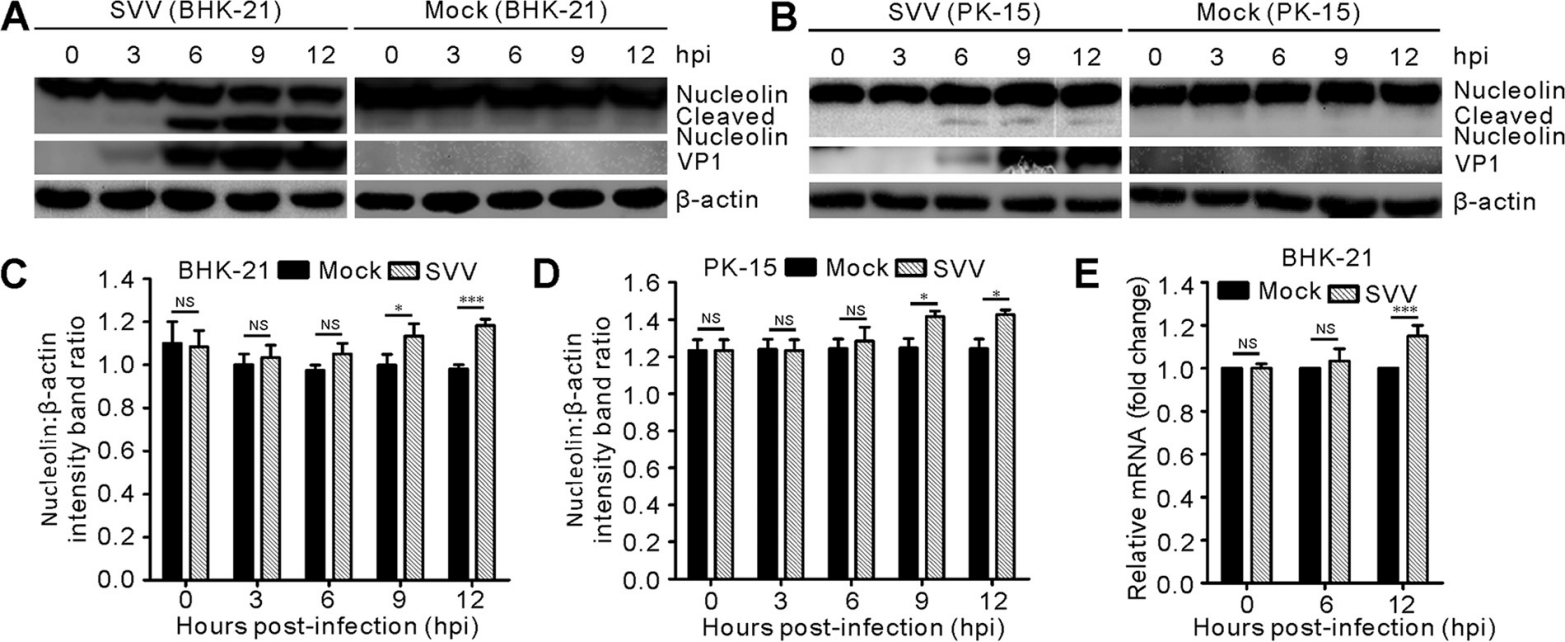
SVV infection cleaved NCL and upregulated NCL expression. (A to B) SVV infected-BHK-21 cells (MOI = 5) and -PK-15 cells (MOI = 5) were collected at 0, 3, 6, 9, and 12 hpi. Cell samples were subjected to Western blotting to analyze the levels of NCL and SVV-VP1. β-actin served as an internal control. (C to D) The expression of NCL was quantified using Image J. Data were analyzed with GraphPad Prism. The error bars indicate mean ± SD from three independent experiments (*, *P < *0.05; ***, *P < *0.001; NS, not significant). (E) BHK-21 cells infected with SVV (MOI = 5) were collected at 0, 6, and 12 hpi. Cell samples were subjected to qRT-PCR to analyze the transcriptional levels of NCL, and β-actin served as an internal control. Statistical analysis was performed using GraphPad Prism. The error bars indicated mean ± SD from three independent experiments (***, *P < *0.001; NS, not significant).

It has been reported that the nucleolus serves as a critical replication compartment for some DNA and RNA viruses (37–39). Given this, we determined the effect of NCL on SVV infection and found that SVV infection induced the cleavage of NCL in BHK-21 cells and PK-15 cells in the early stages of infection, while this protein was not cleaved in mock-infected cells (Fig. 1A and B). In addition, this cleavage effect strengthened over the course of SVV infection (Fig. 1A), suggesting that NCL was cleaved during SVV infection. Subsequent qRT-PCR revealed that NCL transcription was also upregulated in response to SVV infection (Fig. 1E), which was then validated by our Western blot data (Fig. 1A to D, 2B to E). Taken together, these results suggest that SVV infection induces cleavage and upregulation of NCL in cultured cells.

**FIG 2:**
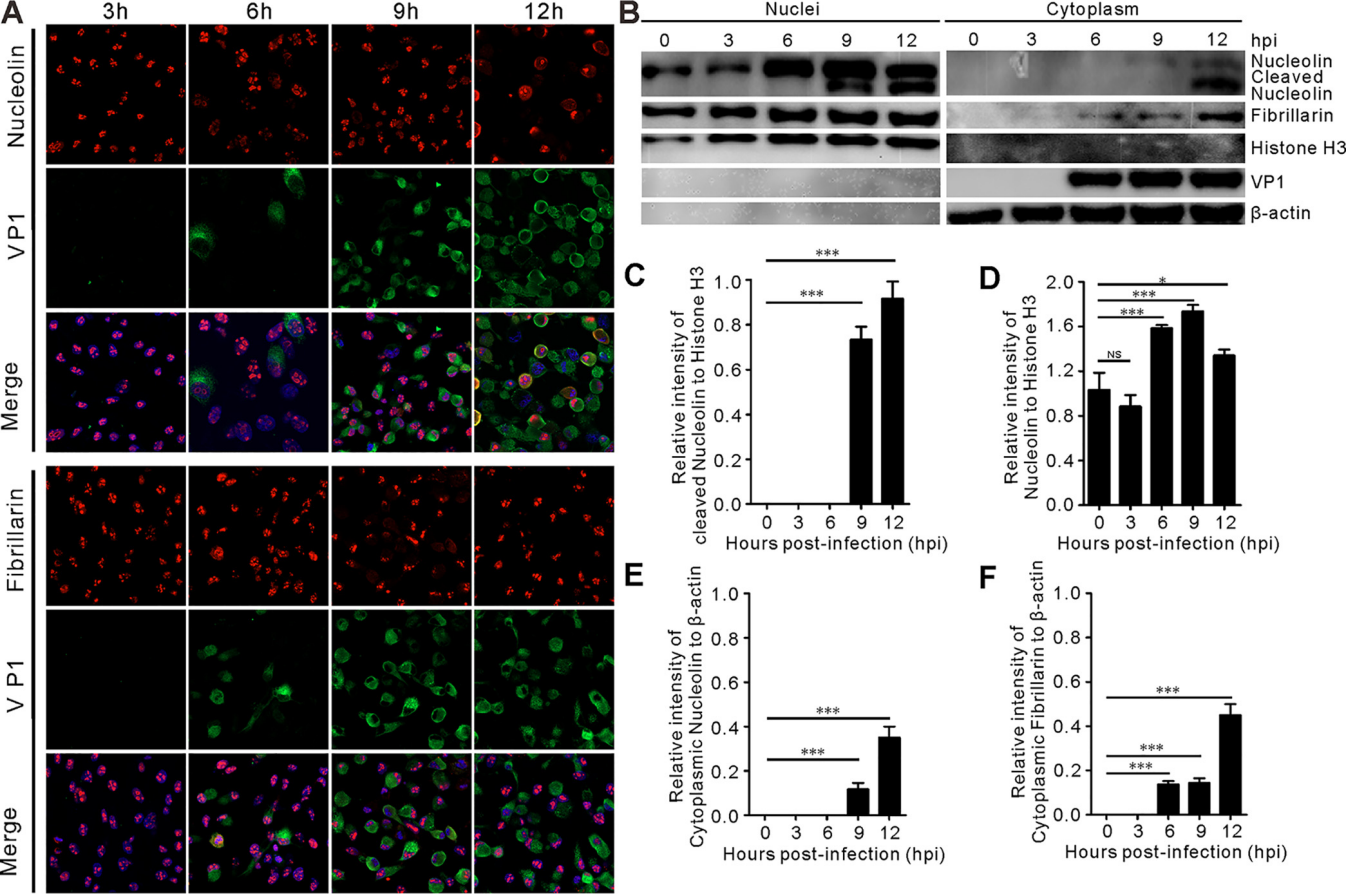
SVV infection induces cytoplasmic redistribution of NCL from the nucleus. (A) BHK-21 cells infected with SVV (MOI = 5) were monitored with fluorescence microscopy to analyze subcellular localization of NCL and fibrillarin at 3, 6, 9, and 12 hpi, respectively. Samples were stained with NCL and fibrillarin antibody (red), VP1 antibody (green), and DAPI (blue), then examined by confocal microscopy. (B) The cell nuclear and cytoplasmic fractions were prepared at 0, 3, 6, 9, and 12 hpi (MOI = 5). The expression of NCL and fibrillarin were by determined Western blotting with antibodies against NCL, fibrillarin, histone H3, and β-actin, VP1 was used as an indicator for SVV infection. (C to D) The graph shows the quantitative analysis of cleaved nuclei NCL normalized against histone H3, and uncleaved nuclei NCL normalized against histone H3, respectively. The quantitative analysis using Image J. Statistical analysis was performed using GraphPad Prism. The error bars indicated mean ± SD from three independent experiments (*, *P < *0.05; ***, *P < *0.001; NS, not significant). (E to F) The relative gray intensity of cytoplasmic NCL and cytoplasmic fibrillarin were normalized against β-actin, respectively. The quantitative analysis using Image J. Statistical analysis was performed using GraphPad Prism. The error bars indicated mean ± SD from three independent experiments (***, *P < *0.001; NS, not significant).

### SVV infection induces translocation of NCL to the cytoplasm.

These findings prompted us to evaluate the relationship between NCL and SVV replication in greater detail. Immunofluorescence assays showed that NCL was predominantly localized to the nucleolus in mock-infected BHK-21 cells (Fig. 2A), but most NCL was redistributed from the nucleus to the cytoplasm in response to SVV infection (Fig. 2A). Similarly, fibrillarin, another important component of nuclei, underwent a similar cytoplasmic relocalization. Given this, we then used cytoplasmic and nuclear fractionation assays to further examine this translocalization in SVV-infected BHK-21 cells (Fig. 2B). The results indicated an increase in the expression of NCL in the nuclei and cytoplasm (Fig. 2B to E), while there was a steady increase in cleaved NCL in the cytoplasm toward the later stages of SVV infection (Fig. 2B and E), which was also shown to occur later than the same phenomenon in the nuclei (Fig. 2B and C), indicating that a course of cleaved NCL transportation is required for viral replication. Although some cleaved nucleolin is found in the cytoplasm, much of which is still found in the nucleus (Fig. 2B). As expected, cytoplasmic redistribution of fibrillarin was significantly enhanced during SVV infection (Fig. 2F). Thus, we were able to confirm the relocalization of NCL to the cytoplasm during SVV infection.

### 3C^pro^ cleaves NCL and induces NCL redistribution.

**FIG 3:**
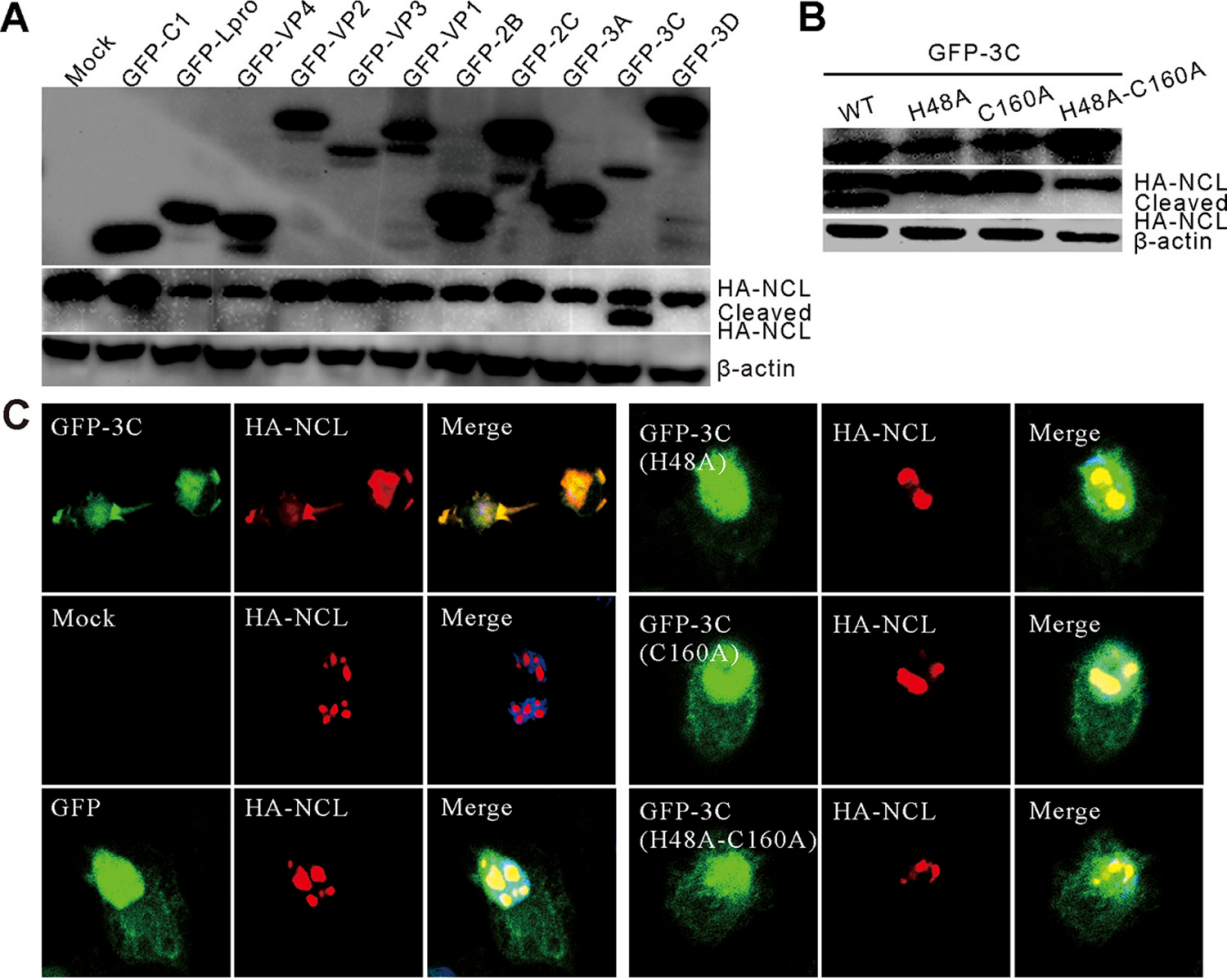
SVV 3C^pro^ was responsible for NCL cleavage and translocation. (A) BHK-21 cells were cotransfected with HA- NCL and GFP-tagged expressing plasmids and subjected to Western blot analysis at 24 h posttransfection with antibodies to GFP, HA, and β-actin served as an internal control. (B) BHK-21 cells were cotransfected GFP-3C, GFP-3C-H48A, GFP-3C-C160A, and GFP-3C-DM (H48A-C160A) and HA-NCL. Samples were subjected to Western blot analysis at 24 h posttransfection with antibodies to GFP tag, HA tag, and β-actin as an internal control. (C) BHK-21 cells were cotransfected GFP, GFP-3C, GFP-3C-H48A, GFP-3C-C160A, and GFP-3C-DM (H48A-C160A) with HA-NCL. At 24 h posttransfection, the cells were stained with antibodies to NCL (red) and DAPI (blue), and examined under confocal microscopy.

We then clarified the mechanism of SVV infection–induced NCL cleavage and redistribution by initially screening for the viral proteins responsible for NCL cleavage and redistribution using Western blotting. Previously constructed SVV protein-expressing plasmids and HA-tagged NCL were co-transfected into BHK-21 cells (17), and our evaluations revealed that only GFP-3C was responsible for cleavage (Fig. 3A), and this *in vitro* cleavage agreed with the cleavage of endogenous NCL in SVV-infected BHK-21 cells (Fig. 1A and B).

Similar to other picornaviruses, SVV 3C^pro^ facilitates the direct cleavage of multiple cellular proteins via its inherent protease activity (12, 13, 18). Plasmids encoding GFP-3C proteins with mutated histidine (H3C48) and cysteine (C3C160) residues, including GFP-3C-H48A, GFP-3C-C160A, and GFP-3C-DM (H48A-C160A), were constructed in our previous study (40) and co-transfected with HA-NCL into BHK21 cells for evaluation. As expected, only wild-type GFP-3C could cleave HA-NCL *in vitro*, as a loss in 3C^pro^ protease activity results in the loss of the NCL cleavage products (Fig. 3B). Therefore, our results provide robust evidence that the conserved protease sites (H3C48 and C3C160) of 3C^pro^ are responsible for the cleavage of NCL.

Finally, the subcellular colocalization of NCL with 3C^pro^ and its mutants was examined using confocal microscopy, which revealed that NCL was predominantly localized to within the nucleolus and that it translocated from the nucleus and redistributed into the cytoplasm only after the co-expression of GFP-3C (Fig. 3C). In contrast, GFP-3C-H48A, GFP-3C-C160A, and GFP-3C-DM did not have any effect on the distribution of HA-NCL (Fig. 3C). Redistribution of nucleolin to the cytoplasm is more likely that the cleavage takes place in the cytoplasm where nucleolin is translated, and some of the cleaved nucleolin fails to enter the nucleus in the first place.

### SVV 3C^pro^ cleaves NCL at residue Q545.

**FIG 4:**
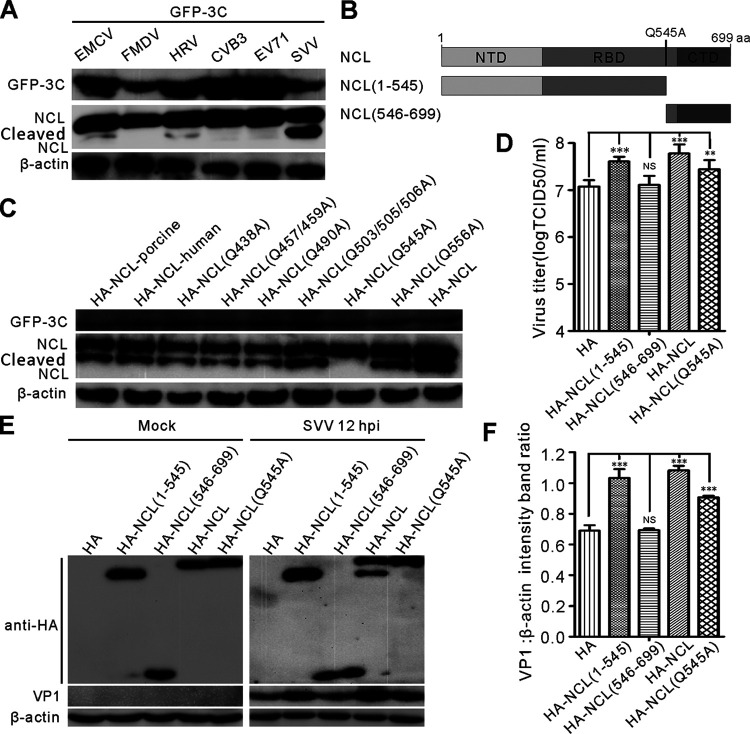
SVV 3C^pro^ targets NCL at residue Q545 for cleavage. (A) BHK-21 cells were cotransfected with GFP-3C^pro^ of EMCV, FMDV, HRV, CVB3, and EV71 with HA-NCL. At 24 h postinfection, and the expression of HA-NCL was analyzed by Western blotting with antibodies to GFP, NCL, and β-actin. (B) Domain organization and engineering of truncation constructs of NCL. NTD, N-terminal domian; RBD, RNA-binding domains; CTD, C-terminal domain. (C) BHK-21 cells were cotransfected with GFP-3C^pro^ with HA-NCL and its mutants. At 24 h postinfection, the level of HA-NCL was analyzed by Western blotting with antibodies to GFP, NCL, and β-actin. (D) The virus productions in HA-NCL (1 to 545), HA-NCL (546 to 699), HA-NCL, HA-NCL (Q545A), and empty HA vectors-transfected BHK-21 cells and control cells at 12 h postinfection were titrated by the endpoint dilution assay. (E) Western blotting examined the VP1 protein production in HA-NCL (1 to 545), HA-NCL (546 to 699), HA-NCL, HA-NCL (Q545A), and empty HA vectors-transfected BHK-21 cells and control cells at 12 h postinfection (MOI = 5). (F) The level of VP1 was quantified normalized against β-actin using Image J. Statistical analysis was performed using GraphPad Prism. The error bars indicated mean ± SD from three independent experiments (*, *P < *0.05; ***, *P < *0.001; NS, not significant).

We further investigated the specificity of NCL cleavage by 3C^pro^ from different picornaviruses, including encephalomyocarditis virus (EMCV), FMDV, human rhinovirus (HRV), coxsackievirus B3 (CVB3), and EV71. We found that EMCV and HRV 3C^pro^ also cleaved NCL; however, the amount of cleavage products was much lower than that of SVV 3C^pro^-cleaved NCL (Fig. 4A). Picornavirus 3C^pro^ preferentially recognizes glutamine-glycine (Q-G) or glutamic acid-glutamine (E-Q) pairs in proteins for cleavage (41). On the basis of molecular weight of cleaved NCL, we generated some mutants within the NCL protein at its Q residues. Thus, a total of eight Q residues (Q438, Q457, Q490, Q503, Q505, Q506, Q545, and Q556) were replaced with alanine (A) (Fig. 4B) and then subjected to 3C^pro^ cleavage. This experiment showed that the specific cleavage pattern for SVV 3C^pro^ disappeared in NCL Q545A, while all eight of the other NCL mutants were still cleaved when treated with SVV 3C^pro^ (Fig. 4C). These results suggest that Q545 is recognized by SVV 3C^pro^ and that this recognition is critical to NCL cleavage. In addition to hamster NCL, both porcine NCL and human NCL were also cleaved by SVV 3C^pro^ *in vitro* (Fig. 4C). Next, we confirmed whether cleavage of NCL potentially affects SVV replication. BHK-21 cells were transfected with HA-NCL (1-545), HA-NCL (546-699), HA-NCL, HA-NCL (Q545A), or empty HA vectors and then infected with SVV before being evaluated using the TCID_50_ assay and Western blot (Fig. 4D and E). These results indicated that both cleaved and intact forms of NCL had a positive role in viral replication (Fig. 4D to F). Consistently, HA-NCL was also cleaved following its transfection into SVV-infected BHK-21 cells, while HA-NCL (Q545A) could not be cleaved (Fig. 4E). Thus, these evaluations revealed that SVV 3C^pro^ cleaves NCL at residue Q545, and cleaved NCL promotes viral replication.

### Silencing NCL inhibits SVV replication.

**FIG 5:**
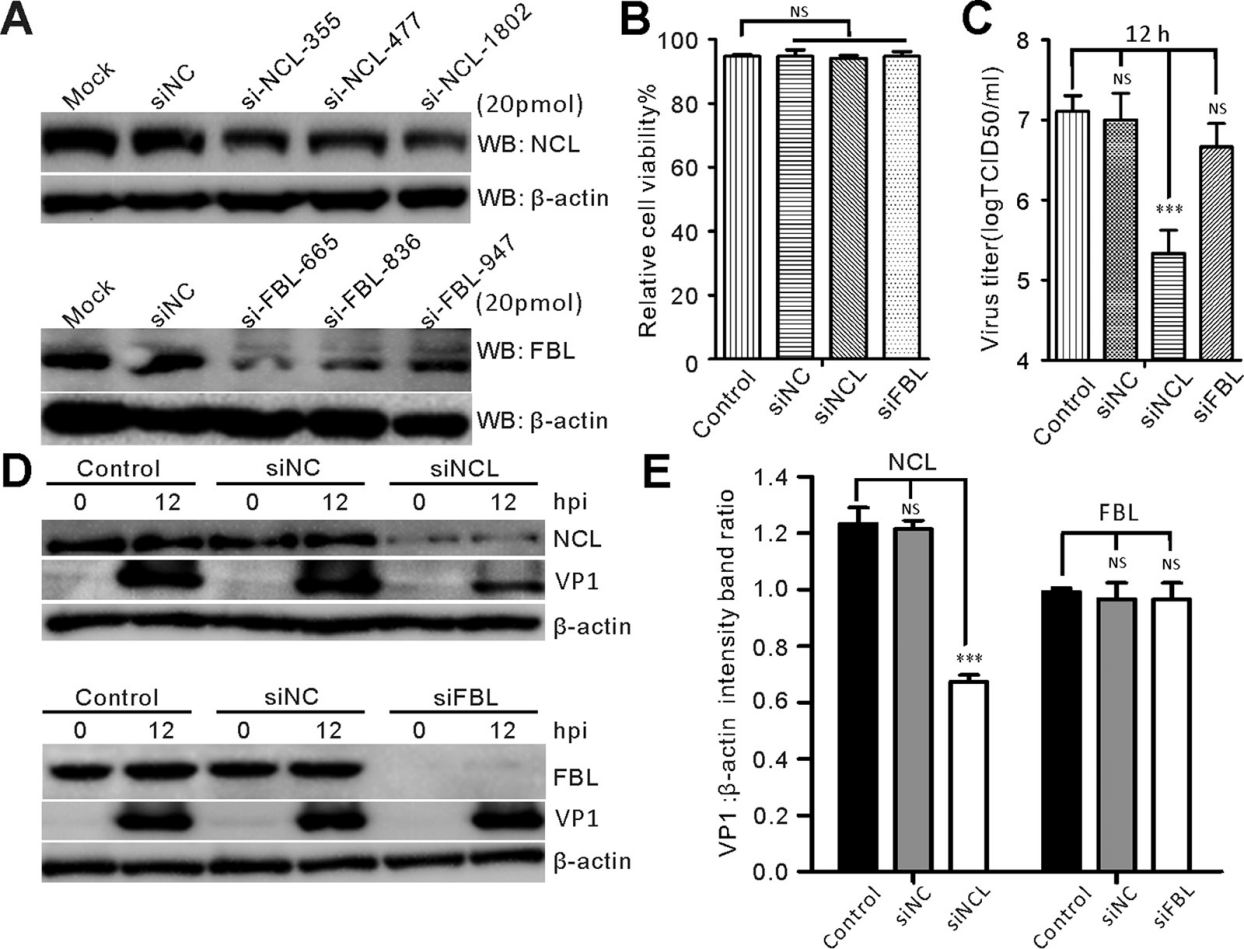
Knockdown of NCL inhibits SVV replication. (A) Western blotting analyzed the expression of NCL and fibrillarin after siRNA-medicated knockdown at a concentration of 20 pmol with specific antibodies. (B) CCK-8 assay analyzed the viability of siRNA-transfected cells and control cells. Statistical analysis was performed using GraphPad Prism. The error bars indicated mean ± SD from three independent experiments (***, *P < *0.001; NS, not significant). (C) The virus productions in siRNA-transfected BHK-21 cells and control cells at 12 h postinfection (MOI = 5) were titrated by the endpoint dilution assay. (D) Western blotting examined the VP1 protein production in siRNA-transfected BHK-21 cells and control cells at 0 h and 12 h postinfection (MOI = 5). (E) The expression of VP1 was quantified normalized against β-actin using Image J. Statistical analysis was performed using GraphPad Prism. The error bars indicated mean ± SD from three independent experiments (*, *P < *0.05; ***, *P < *0.001; NS, not significant).

We continued to examine the impact of NCL on SVV replication by treating BHK-21 cells with three different NCL small-interfering RNAs (siRNAs) and a negative control (NC) siRNA, while fibrillarin knockdown was used as a control. Western blot analysis showed efficient knockdown at 20 pmol siRNA when compared with mock-transfected cells and siNC-transfected cells (Fig. 5A). Cell viability assays indicated that knockdown of NCL and fibrillarin had no effect on cellular proliferation (Fig. 5B) but silencing of NCL (si-NCL-1802) significantly inhibited SVV infection, as evidenced by changes in the TCID_50_ assays (Fig. 5C). The viral yields greatly decreased following NCL (si-NCL-1802) knockdown, as revealed by decreases in the viral titer at 12 h postinfection (Fig. 5C), and viral VP1 protein production showed a dramatic decrease in response to reduced NCL expression when compared with both the mock-transfected and siNC-transfected cells (Fig. 5D and E). In contrast, fibrillarin knockdown had no effect on virus titer or viral protein production (Fig. 5C to E). Collectively, these results indicate that NCL is closely associated with SVV replication.

### Overexpression of NCL promotes SVV replication.

**FIG 6:**
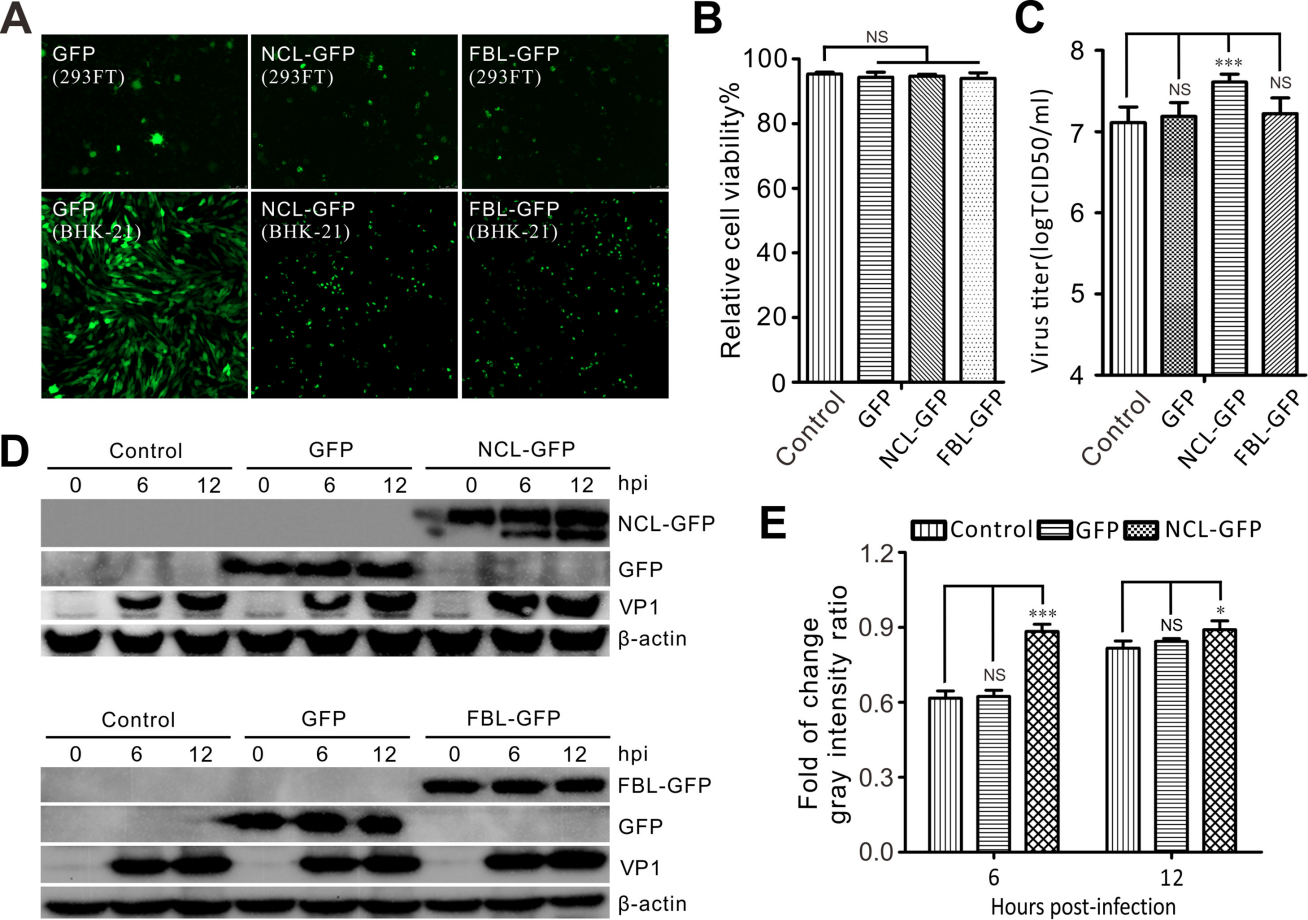
Upregulation of NCL promotes SVV replication. (A) Fluorescence of packed lentiviruses in HEK-293FT cells, and lentivirus-transduced BHK-21 cells expressing GFP, NCL-GFP, and FBL-GFP were captured with fluorescence microscopy at 24 h posttransfection and transduction. (B) Cell viability of lentivirus transduced BHK-21 cells were determined by CCK-8 assay (NS, not significant). (C to D) BHK-21 cells were transduced with lentivirus expressing GFP, NCL-GFP, and FBL-GFP. Cells were infected with SVV (MOI = 5) after 48 h postransduction, and the expression of VP1 was analyzed by TCID_50_ assay and Western blotting with antibodies to GFP, VP1, and β-actin at 0, 6, and 12 hpi, respectively. Statistical analysis was performed using GraphPad Prism. The error bars indicated mean ± SD from three independent experiments (*, *P < *0.05; ***, *P < *0.001; NS, not significant). (E) The expression of VP1 was quantified normalized against β-actin using Image J. Statistical analysis was performed using GraphPad Prism. The error bars indicated mean ± SD from three independent experiments (*, *P < *0.05; ***, *P < *0.001; NS, not significant).

Given that the knockdown of NCL inhibited SVV replication, we then went on to evaluate the effect of NCL overexpression on the proliferation of SVV using a lentiviral system. Lentiviruses expressing NCL-GFP, fibrillarin-GFP, and GFP were rescued in HEK293FT cells (Fig. 6A) and stable BHK-21 cell lines overexpressing NCL-GFP, fibrillarin-GFP, and GFP were established using lentiviral transduction (Fig. 6A). Both viability and cell growth in these lentivirus-transduced cells were similarly to those of the non-transduced cells (Fig. 6B), but viral VP1 protein production and viral titer both demonstrated a dramatic increase in NCL-GFP expressing cells when compared with the controls (Fig. 6C and D). Overexpression of fibrillarin had no effect on viral VP1 protein production or viral titer (Fig. 6C and D), which was consistent with the results from the fibrillarin knockdown assays. Taken together, these results demonstrated that overexpression of NCL enhances SVV propagation.

## DISCUSSION

The nucleolus is a subnuclear cellular compartment, and NCL, nucleophosmin, and fibrillarin are the major protein components of the nucleoli, which act as sites for ribosome RNA biogenesis and RNA transcription. NCL consists of three multifunctional domains including a highly phosphorylated N-terminal domain, a central RNA-binding domain, and a glycine/arginine-rich C-terminal domain (42). An increasing number of studies have revealed that numerous viruses hijack the nucleolar proteins for their own replication (28–30, 43, 44) and the nucleolar-cytoplasmic redistribution of NCL plays an important role in the life cycle of several picornaviruses (29, 30, 36). Here, we found that NCL was cleaved and translocated (Fig. 1A and 2B) and that the expression of this cleaved NCL progressively increased over the course of SVV infection (Fig. 1A and B). We also found that the mRNA levels of NCL increased at the later stages of infection (Fig. 1C), which is similar to some observations recorded for NCL upon HSV-1 infection (39). SVV replication cycle is complete in 9 h in both BHK-21 and PK-15 cultured cells (Fig. 1A and B). In general, the relevance of NCL mRNA upregulation is finished after the infectious cycle. However, we still observed the NCL mRNA increase in 12-h samples (Fig. 1A and B), this likely reflects the difference of the RNA isolation from live and dead cells at 12 hours postinfection (hpi). Both Western blotting and immunofluorescence assay showed that NCL translocated to the cytoplasm from the nucleoli (Fig. 2A); however, fibrillarin, another nucleolar protein, remained in the nucleolus following SVV infection, with only a minor proportion relocalized to the cytoplasm (Fig. 2A). These results were similar to those of poliovirus-infected cells, which only induced NCL redistribution but not fibrillarin redistribution (30). This indicates the specificity of the translocation of nucleolar proteins and their specific functional connection to the infectious agent. Confocal microscopy and nuclear and cytoplasmic fractionation assays also confirmed that cleavage and redistribution of NCL was induced by SVV infection. The expression of NCL increased in the nuclei after SVV infection, with this being most obvious at 6 h postinfection (Fig. 2B). The cleaved NCL was greatly enhanced in the nuclei and cytoplasmic fractions at the later stages of SVV infection (Fig. 2B and C) and the expression of fibrillarin in the nuclei and cytoplasm also gradually increased over the course of SVV infection (Fig. 2B, E, and F), although this remained less significant than the increases associated with NCL. This suggests preferential translocation of NCL. The causes of this redistribution are not yet well understood, and future work should be focused on evaluating the retention of cytoplasmic NCL as this may reveal its specific contribution to SVV replication.

Further investigation revealed that SVV 3C^pro^ was responsible for the cleavage and redistribution of NCL, and Western blot mediated screening of the SVV proteins verified that only 3C^pro^ cleaved NCL (Fig. 1A and 3A). When any of the catalytic residues of 3C^pro^ were mutated, cleavage was abolished (Fig. 3B) and only wild-type 3C^pro^ induced the redistribution of NCL. Similar results were observed for rhinovirus 3C^pro^ cleavage of NCL in infected host cells (45). FMDV 3C^pro^ also induced the redistribution of NCL, with this redistribution shown to be dependent on proteinase activity, but this protease did not cleave NCL (29). We found that 3C^pro^ from EMCV and HRV cleave NCL, while 3C^pro^ from FMDV, CVB3, and EV71 did not (Fig. 4A), all of which was consistent with previous studies (45). A similar evaluation revealed that neither mouse GSDMD nor human GSDMD was cleaved by SVV 3C^pro^; only porcine GSDMD was targeted for cleavage (18). However, we found that SVV 3C^pro^ cleaved porcine NCL and cleaved human NCL (Fig. 1A and 4C) suggesting that the cleavage of specific cellular proteins is dependent on the cell and virus type being evaluated. Picornavirus 3C^pro^ preferentially cleaves Q-G or E-Q pairs (41), prompting us to mutate each of the nine Q residues to A residues in NCL, it is worth noting that there were no E-Q pairs in this protein, so this was not repeated for those recognition sites. Interestingly, our results revealed that only one of the two Q-G pairs in NCL was the target cleavage site, with the replacement of Q-G (545-546) with A-G (545-546) completely abolishing SVV 3C^pro^ mediated cleavage of this Q545A NCL (Fig. 4C). In addition, we found that the SVV 3C^pro^ cleavage sites of various cellular factors were similar to those reported in previous studies (12, 13, 16, 18). We also revealed that cleaved NCL (aa.1-545) contributed to SVV replication (Fig. 4D to F), and we hypothesized that it may interact with viral proteins or the viral genome to regulate viral replication, but these speculations require further study.

However, this is in agreement with previous reports that suggest that NCL enhances viral protein function and infectious progeny yields (28, 29, 34, 39, 43, 44, 46). NCL interacts with human cytomegalovirus (HCMV) UL44 often colocalizing with UL44 at the periphery of replication compartments inducing the improper localization of UL44 and HCMV DNA to facilitate synthesis (44, 47). HCMV UL84 localizes with both the viral DNA and NCL within the subnuclear replication compartments (37). Conserved residues in HSV-1 UL24 are crucial for dispersal of the NCL (38) forcing it to relocalize outside of the nucleoli and colocalize with ICP8 in the viral replication compartments used by HSV-1 during pathogenesis (39). NCL knockdown influences the transportation of US11 between the nucleolus and the cytoplasm, and NCL silencing suggests that this protein is required for HSV-1 replication (39). In addition, NCL expression is essential for nuclear egress of HSV-1 nucleocapsids (43), and EV71 VP1 capsid proteins directly interact with NCL, and downregulation of cell surface NCL expression reduces viral binding, infection, and yield, while its upregulation promotes viral binding, infection, and yield (36).

Cell surface NCL plays an important role in viral internalization and entry and is involved in EV71 entry via its interactions with the VP1 capsid of this virus (36, 48). Influenza A virus hemagglutinin interacts with cell surface NCL, facilitating viral internalization and entry (49) while the RHDV capsid protein interacts with NCL to facilitate viral internalization via clathrin-dependent endocytosis (31). The fusion envelope glycoprotein of RSV also interacts with the NCL cell surface receptor, which has been shown to be required for viral entry (32, 50). Cell surface NCL was also identified as a putative Crimean-Congo hemorrhagic fever virus entry factor (51) and is essential for human parainfluenza virus type 3 (HPIV-3) internalization in human lung epithelial A549 cells. In addition, treatment with anti-NCL antibodies and purified NCL significantly inhibited HPIV-3 replication (52). Thus, targeting cell surface NCL may provide several novel avenues for the effective treatment of several infectious diseases, making it interesting to investigate the role of NCL in the internalization and entry of SVV in the future, as we also observed that NCL translocated into the replication compartments and colocalized with the SVV VP1 protein (Fig. 2A).

NCL also interacts with the IRES of FMDV, which regulates viral IRES-driven translation, and NCL knockdown significantly inhibits FMDV production (29). The P-protein of rabies virus localizes to nucleoli and interacts with NCL, and the depletion of NCL expression inhibits viral growth (46). In contrast, NCL inhibits the replication of peste des petits ruminants virus (53). Here, we used siRNA-mediated knockdown and lentiviral-mediated overexpression constructs to evaluate the potential involvement of NCL in SVV replication. Our data suggest that NCL depletion dramatically reduced the production of infectious viral particles and viral protein production, while NCL upregulation markedly enhanced SVV replication (Fig. 5 and 6). These results suggest that NCL is a pivotal nucleolar protein closely associated with replication in picornaviruses.

In summary, our findings reveal the cleavage and translocation of NCL in SVV-infected cells, and that the protease activity of SVV 3C^pro^ is sufficient to cleave NCL and relocalize it to the cytoplasm. In addition, we demonstrate that SVV 3C^pro^ cleaves NCL at residue Q545, demonstrating the likely contribution of NCL to SVV replication. Taken together, these results provide further insight into the mechanisms by which SVV utilizes nuclei for efficient replication.

## MATERIALS AND METHODS

### Cells, viruses, and antibodies.

BHK-21 cells and PK-15 cells were grown in Dulbecco’s modified Eagle’s medium (DMEM) (Invitrogen, CA, USA) containing 10% fetal bovine serum (FBS) (Invitrogen) at 37°C in a humidified atmosphere with 5% CO_2_. The SVV strain CHhb17 has been documented previously (54). Mouse monoclonal antibody against VP1 were produced by our lab and used as previously described (54). Rabbit anti-nucleolin (14574S) was obtained from Cell Signaling Technology (Beverly, MA, USA). Rabbit anti-fibrillarin (A13490) and mouse anti-GFP (AE012) were obtained from ABclonal (Wuhan, China). Rabbit anti-histone H3 antibody (ab183902) and mouse anti-β-actin antibody (ab8226) were obtained from Abcam (Cambridge, MA, USA). Anti-rabbit IgG, horseradish peroxidase (HRP)-linked antibody (7074) and anti-rabbit IgG, HRP-linked antibody (7046) were obtained from Cell Signaling Technology. Alexa-568-conjugated goat anti-rabbit (11011) and alexa-488-conjugated goat anti-mouse (11017) were obtained from Invitrogen.

### Plasmid construction.

The nucleolin gene was amplified and constructed into the vector pCMV-HA (Clontech, 631604) and vector pWPXL (Addgen, 12257) by using ClonExpress One Step Cloning Kit (Vazyme, China). GFP-fused SVV structural and nonstructural proteins were recombined into the vector pEGFP-C1 (Clontech, U55763) that has been constructed in our previous studies (17). The single point and double points mutants of GFP-3C, such as GFP-3C-H48A, GFP-3C-C160A, GFP-3C-DM (H48A and C160A double mutants), and mutants of HA-NCL-Q438A, HA-NCL-Q457A-Q459A, HA-NCL-Q490A, HA-NCL-Q503A-Q505A-Q506A, HA-NCL-Q545A, HA-NCL-Q556A were performed with mutagenesis polymerase chain reaction (PCR). The 3C sequence of EMCV, FMDV, Rhinovirus, CVB3, and EV71 were synthesized from Tsingke Biotechnology (Beijing, China) and recombined into the vector pEGFP-C1. Plasmids were transfected using Lipofectamine 3000 (Thermo Fisher, L3000015). Primers used for plasmid construction are listed in Table 1.

**TABLE 1:** Primers used in this study

Primers^*a*^	Sequence (5′–3′)^*b*^	Restriction site
HA-NCL-F HA-NCL-R HA-NCL (546-699)-F HA-NCL (1-545)-R NCL-GFP-F NCL-GFP-R Fibrillarin-GFP-F Fibrillarin-GFP-R	TGGCCATGGAGGCCCGAATTCGGATGGCTCCTCCTCCAAAGGAGGTG GATCCCCGCGGCCGCGGTACCTTATTCAAACTTCGTCTTCTTTCC TGGCCATGGAGGCCCGAATTCGGGGACCCAGGGGATCGCCTAATG GATCCCCGCGGCCGCGGTACCTTATTGTAACTCCAGCCTGATTGT GAGGTTTAAACTACGGGATCCAATGGCTCCTCCTCCAAAGGAGGTG ACCGGTAGCGCTAGGACGCGTAATTCAAACTTCGTCTTCTTTCCT GAGGTTTAAACTACGGGATCCAATGAAGCCAGGTTTCAGCCCCC ACCGGTAGCGCTAGGACGCGTAAGTTCTTCACCTTCGGAGGCGG	EcoRI KpnI EcoRI KpnI BamHI MluI BamHI MluI

a F denotes forward primer; R denotes reverse primer.

b Restriction sites are underlined.

### Western blotting.

Cells were collected and lysed using lysis buffer containing 0.5% NP-40, 50 mM Tris, 0.5 mM EDTA, 150 mM NaCl and protease inhibitor. The protein concentration of cell lysates was quantified using the bicinchoninic acid kit (Thermo Fisher, 23225). Equal amounts of protein samples were separated by sodium dodecyl sulfate polyacrylamide gel electrophoresis (SDS-PAGE) and transferred to nitrocellulose (NC) membranes (PALL, FL, USA), then blocked with phosphate-buffered saline (PBS) buffer containing 5% nonfat milk. Membranes were probed with primary antibodies for 4 h, then washed with PBST (PBS with 0.05% Tween 20) and incubated with HRP-conjugated secondary antibodies. Membrane-bound antibodies were detected using enhanced chemiluminescence detection reagents (Thermo Fisher, 34096).

### Quantitative reverse transcription-PCR (qRT-PCR).

Total RNA was extracted from cells using TRIzol reagent (Invitrogen). The first strand cDNA was synthesized using 0.5 μg total RNA with RT SuperMix for qPCR (R122) (Vazyme, China), and qRT-PCR using SYBR green Master Mix (Vazyme, Q111). The 2^-ΔΔCT^ method was used to calculate the relative fold change. The mRNA level of β-actin served as the reference control. The primer sequences for hamster NCL: (forward primer 5′-GTTCGAGCTGCAAGAACACT-3′ and reverse primer 5′-CTCTGCATCAGCTTCGGACT-3′), and hamster β-actin (forward primer 5′-GTAGCCATTCAGGCCGTGCT-3′ and reverse primer 5′-ATGGCATGAGGGAGAGCGT-3′) were used.

### Immunofluorescence assay.

Immunofluorescence assays (IFA) were performed as described in our previous studies (40). Briefly, cells were fixed with 4% paraformaldehyde for 10 min and permeabilized with 0.1% Triton X-100 for 10 min, then blocked with 2% bovine serum albumin (BSA) in PBS. Cells were incubated with the specific primary antibodies for 1 h. After washing with PBS, cells were incubated with the specific secondary antibodies for another 1 h, then incubated with 4’,6-diamidino-2-phenylindole (DAPI). Images were taken under a Nikon A1 confocal microscopy.

### Nuclear and cytoplasmic fractionation.

BHK-21 cells infected with SVV were harvested at indicated times postinfection, and nuclear and cytoplasmic fractions were fractionated using the nuclear and cytoplasmic fractionation kit (Thermo Fisher, 78833) in accordance with the manufacturer’s instructions. The nuclear and cytoplasmic samples were subjected to Western blotting, β-actin and histone-H3 served as internal references for cytoplasmic and nuclear proteins, respectively.

### Lentivirus packaging.

The lentivirus system containing three plasmids were obtained from Addgene. 1.18 μg pWPXL, 0.47 μg pMD2.G, and 2.35 μg psPAX2 were co-transfected into HEK293FT cells. At 24 h posttransfection, the supernatants were harvested and concentrated. BHK-21 cells were transduced with lentiviruses in the presence of Polybrene. At 48 h postransduction, BHK-21 cells were incubated with SVV.

### Virus infection and titration.

After incubation with SVV for 1 h at 37°C, BHK-21 cells were washed three times with DMEM and then supplemented with DMEM medium containing 2% FBS. The cultured medium was harvested at the indicated times after SVV infection. Samples were titrated on BHK-21 cells using limiting dilution assay and represented as the tissue culture infectious dose 50 (TCID_50_) (17).

### RNA interference.

The small interfering RNAs (siRNAs) targeting NCL and fibrillarin in three different coding regions were synthesized in GenePharma (Suzhou, China): si-NCL-355 (sense, 5′-GCGAAAGCAUUGGUAGCAATT -3′; antisense, 5′-UUGCUACCAAUGCUUUCGCTT-3′), si-NCL-477 (sense, 5′-GGAUGAAGAUGACAGUGAUTT-3′; antisense, 5′-AUCACUGUCAUCUUCAUCCTT-3′), si-NCL-1802 (sense, 5′- GCUCUGUUCGUGCAAGAAUTT-3′; antisense, 5′- AUUCUUGCACGAACAGAGCTT -3′), si-FBL-665 (sense, 5′- CCCGGACGGUCUGGUCUAUTT -3′; antisense, 5′- AUAGACCAGACCGUCCGGGTT -3′), si-FBL-836 (sense, 5′- GCCAGACCAGACUCGGAUUTT -3′; antisense, 5′- AAUCCGAGUCUGGUCUGGCTT -3′), si-FBL-947 (sense, 5′- GGCCGUGUUUGCCUCUGAATT -3′; antisense, 5′- UUCAGAGGCAAACACGGCCTT -3′). The siRNA (sense, 5′-UUCUCCGAACGUGUCACGUTT-3′; antisense, 5′-ACGUGACACGUUCGGAGAATT-3′) served as a negative control. siRNAs were transfected using Lipofectamine RNAiMAX (Thermo Fisher, 13778150). Cells were harvested at 48 h posttransfection, the expression levels of NCL and FBL were subjected to Western blotting, and cell viability of gene-silenced cells was detected by CCK-8 assay. At 48 h posttransfection, cells were infected with SVV.

### Statistical analysis.

All data were evaluated with GraphPad Prism (La Jolla, CA, USA). Error bars indicate means ± standard deviations (SD). *P*-values are indicated using asterisks, with a P < 0.05 was considered statistically significant.
